# Reducing the Cut-Off Value of the Fecal Immunochemical Test for Symptomatic Patients Does Not Improve Diagnostic Performance

**DOI:** 10.3389/fmed.2020.00410

**Published:** 2020-09-02

**Authors:** Mercedes Navarro, Gonzalo Hijos, Carlos Sostres, Alberto Lué, Juan Jose Puente-Lanzarote, Patricia Carrera-Lasfuentes, Angel Lanas

**Affiliations:** ^1^Digestive Diseases Service, University Clinic Hospital, Zaragoza, Spain; ^2^Clinical Chemistry Laboratory, University Clinic Hospital, Zaragoza, Spain; ^3^CIBER Enfermedades Hepáticas y Digestivas (CIBERehd), Zaragoza, Spain; ^4^Department of Medicine, University of Zaragoza, Zaragoza, Spain; ^5^IIS Aragón, Zaragoza, Spain

**Keywords:** colorectal cancer, faecal immunochemical test (FIT), symptomatic patients, cut off value, colonoscopy, colorectal adenocarcinoma

## Abstract

**Introduction:** The fecal immunochemical test (FIT) has been established as a cost-effective test in colon cancer screening programmes. This test could also be helpful in symptomatic patients prior to colonoscopy, but data about diagnostic performance, and accurate cut-off values for these patients are still scarce.

**Materials and Methods:** Prospective study that included consecutive unselected patients with gastrointestinal symptoms referred for colonoscopy between November 2016 and June 2018. We performed a FIT (FOB Gold^®^ test, cut-off 20 micrograms of Hb/gram of feces) prior to colonoscopy and determined the accuracy of FIT in terms of sensitivity, specificity, positive and negative predictive value for clinically significant pathology, advanced neoplasia, and colorectal cancer in symptomatic patients, using two different cut-off values.

**Results:** A total of 727 patients (44.3% men, aged 58.5 ± 14.9 years) was included in the study. The main symptom was history of previous (non-active) rectal bleeding (34.7%), followed by diarrhea (15.0%). Over one quarter of the patients (25.9%) had a positive FIT result. The caecal intubation rate was 95.5%. Clinically significant pathology was identified in 142 colonoscopies (19.5%), advanced neoplasia in 115 (15.8%) and colorectal cancer in 36 colonoscopies (5.0%). FIT performed very well for clinically significant pathology, advanced neoplasia and cancer, with a high negative predictive value (NPV). Reducing the cut-off value to 10 μg/g yielded similar NPV results, with a decrease in specificity. Using a combination of symptoms with a positive FIT result did not improve FIT performance. Only specificity was slightly higher compared to FIT alone, but this was paralleled by a decrease in sensitivity and NPV for cancer and clinically significant pathology. The odds of presenting clinically significant pathology, advanced neoplasia, or cancer increased with FIT concentration.

**Conclusions:** The specificity and NPV of FIT for clinically significant pathology, advanced neoplasia, and cancer are high in symptomatic patients. FIT is a helpful test for determining the need to perform further studies. It may not be necessary to reduce the cut-off value for symptomatic patients, since FIT performance with the current standard cut-off value used in colorectal cancer screening was accurate. FIT can be used to avoid or prioritize colonoscopy procedures.

## Introduction

Colorectal cancer (CRC) is still one of the most commonly diagnosed cancers around the world, being the second most prevalent type in women and the third in men (1). Multiple population-based screening programmes have been implemented in the past few years, based mostly on the fecal immunochemical test (FIT) (2, 3), which appears to be cost-effective compared to non-screening (4, 5).

Gastrointestinal symptoms such as rectal bleeding, change in bowel habits, diarrhea, or abdominal pain are common to different gastrointestinal diseases and are considered poor predictors of the risk of CRC (6). FIT is becoming more widely used in clinical practice as a diagnostic test for the evaluation of symptomatic patients before colonoscopy. In the past few years, several studies (7–10) on the diagnostic accuracy of FIT in this setting have been published, with promising results, but data are still scarce.

As FIT is a quantitative test, the cut-off value can be chosen to adapt each local programme to the availability of endoscopic resources (11, 12). Some studies suggest that fecal hemoglobin concentration could be a predictor of risk for advanced neoplasia and CRC and could be used to prioritize the use of colonoscopy in symptomatic and asymptomatic patients who have a higher risk of presenting CRC or advanced neoplasia (13–17).

Along the same line, NICE Guidance (18) and other recent clinical practice guidelines (19) recommend the use of FIT for the evaluation of symptomatic patients, but with a cut-off value lower than that usually used in CRC screening programmes (10 vs. 20 μg/g)^2^. This could result in a higher rate of false positive tests, with an increase in the demand for colonoscopies, which could lead to longer waiting times for patients and a potential negative impact on patients' prognosis (20, 21).

In this study, we seek to determine the diagnostic performance of FIT in patients with gastrointestinal symptoms in terms of sensitivity, specificity, positive and negative predictive value for CRC, advanced neoplasia, and clinically significant pathology.

## Materials and Methods

### Study Population

This prospective observational study included patients referred to a general tertiary hospital between November 2016 and June 2018 for colonoscopy due to gastrointestinal symptoms such as prior history of rectal bleeding, abdominal pain, change in bowel habits, or diarrhea. We asked these patients to undergo a fecal immunochemical test prior to starting bowel preparation for colonoscopy. All patients included in the study signed an informed consent form, and the study was approved by the Regional Ethical Committee of Aragón (CEICA).

Patients referred for colonoscopy because of CRC screening or adenoma follow-up were excluded. Other exclusion criteria were as follows: presence of comorbidities that contraindicated the use of colonoscopy, personal history of CRC, colectomy or a prior diagnosis of inflammatory bowel disease, family history of at least one first-degree relative diagnosed with CRC or family history suggesting a hereditary family syndrome.

Positive and negative FIT patients were included in the study.

### Fecal Immunochemical Test

Patients were instructed on how to collect a fecal sample according to the written instructions given with the commercial kit, which included no medication or dietary restrictions. The fecal material was collected in a sampling tube and analyzed using FOB Gold^®^ (SENTiFIT; Sysmex-Sentinel CH. SpA, Barcelona, Spain). The cut-off value applied was 117 ng/ml of buffer (equivalent to 20 mg of Hb per gram of feces). For patients referred due to rectal bleeding, this symptom should have stopped at least 48 h prior to the performance of the test, which was assessed by a phone call interview.

### Colonoscopy, Histologic Examination, and Definitions

The colonoscopies were performed by experienced gastroenterologists from the Digestive Diseases Service of our center. Any polypoid lesions detected during the procedure were removed and classified according to the Spanish Network of Cancer Screening Programmes, based on the European guidelines for quality assurance in colorectal cancer screening and diagnosis (22), by an experienced pathologist. Advanced neoplasia is defined by the European Society of Gastrointestinal Endoscopy (ESGE) (23) as the presence of colorectal cancer or a colorectal adenoma with villous histology, high-grade dysplasia, or ≥10 mm in size. Tumor staging was established according to the TNM classification system of the Union for International Cancer Control (24). The term “Any lesion” used in this study refers to the presence of any finding in the colonoscopy (non-adenomatous polyp, non-advanced adenoma, advanced adenoma, CRC, inflammatory bowel disease, or vascular pathology). “Clinically significant pathology” was defined as the presence of CRC, advanced adenoma, inflammatory bowel disease, or vascular pathology such as angiodysplasia.

In this study, we considered right-sided lesions to be those found in the caecum, ascending colon, hepatic flexure, or transverse colon. Left-sided lesions included those found in the sigmoid, descending colon, or splenic flexure. Rectal lesions were analyzed alone and in combination with left-sided lesions.

### Endpoint of the Study

The primary endpoint was to evaluate the diagnostic performance of the fecal immunochemical test according to the parameters of sensitivity (Se), specificity (Sp), and positive and negative predictive values (PPV, NPV) for different colorectal lesions: CRC, advanced neoplasia (CRC or advanced adenoma), and clinically significant pathology. The secondary endpoints were:

- To evaluate the diagnostic performance of FIT using different cut-off values.
- To evaluate the detection rate of the different lesions described above.
- To evaluate the diagnostic performance of FIT in combination with different symptoms.

### Statistical Analysis

First, we performed a descriptive analysis of the population included in the study. Continuous variables were reported as mean with standard deviation (SD), whereas qualitative variables were expressed as frequencies and percentages. In order to evaluate the diagnostic performance of positive FIT alone and in combination with symptoms, we calculated the sensitivity (Se), specificity (Sp), positive predictive value (PPV), negative predictive value (NPV), and diagnostic odds ratio (OR) with 95% confidence intervals (CI) for different colorectal lesions. Numbers needed to treat (NNT) to detect a CRC, advanced neoplasia, and clinically significant pathology were calculated. A logistic regression analysis was performed to determine the association of FIT quartiles with the detection of advanced neoplasia and clinically significant pathology, ORs (95%CI) adjusted by sex and age were reported. We determined positive FIT using the standard cut-off value (20 μg/g) and the cut-off value suggested in some guidelines (10 μg/g). A stratified analysis according to sex and age was also made; potential interactions between FIT and demographic variables were assessed by the correspondingly regression models containing the interaction term. To compare the diagnostic accuracy between 20 and 10 μg/g cut-off, the *DTComPair* package implemented in R was used, the McNemar's test was applied to compare the sensitivities and specificities, and the generalized score statistic proposed by Leisenring, Alonzo and Pepe (2000) was applied for evaluating the differences in (positive and negative) predictive values. A two-sided *p* < 0.05 was considered statistically significant. The statistical analysis was performed using the SPSS software v. 26.0 for Windows (SPSS Ibérica, Madrid, Spain).

### Sample Size Calculation

We calculated the sample size for this study based on the results of a pilot study of the group carried out with 100 patients, which obtained a CRC prevalence of 8%, and a FIT sensitivity of 100%, and specificity of 83.7%. A sample size of 633 patients would be necessary to estimate the sensitivity and specificity of the test with an accuracy of 3% and a confidence level of 95%.

## Results

### Study Population

Eight-hundred and sixty unselected patients were initially contacted and 776 eventually participated in the study. Thirty five patients were additionally excluded due to family history of CRC whereas other 14 patients had two procedures during the study period. A total of 727 patients were included in the final analysis. More women than men participated (55.7% women; 405). The mean age of patients was 58.5 ± 14.9 years old, with the youngest being 18 and the eldest, 89 years old.

The main indication for colonoscopy was a prior history of rectal bleeding (34.7%), followed by diarrhea (15.0%) (Table 1); 14.6% (106) of patients were taking antiplatelet agents and 3.2% (23) anticoagulants. Caecal intubation was achieved in 95.5% (694) of the colonoscopies. The Boston Bowel Preparation Scale was higher than 6 in 85.2%.

**Table 1:** Indications for colonoscopy.

	***n* (%)**
Prior history of rectal bleeding	252 (34.7)
Diarrhea	109 (15.0)
Change in bowel habits	108 (14.9)
Abdominal pain	101 (13.9)
Anemia	87 (12.0)
Others	35 (4.8)
Constipation	21 (2.9)
Constitutional symptoms	14 (1.9)
**Total**	**727 (100.0)**

A lesion was found in 35.9% of patients (261/727), there was clinically significant pathology in 19.5% (142/727) and advanced neoplasia was found in 15.8% (115/727). Cancer was found in 5.0% of patients (36/727) (Tables 2, 3). The cancer was located in the rectum in 36.1% (13) of cases, in the left-sided colon in 33.3% (12) and in the right-sided colon in 30.6% (11). 24.35% (28) of cases of advanced neoplasia were detected in the rectum, 27.8% (32) in the right-sided colon and 47.8% (55) in the left-sided colon.

**Table 2:** Colonoscopy findings.

	***n* (%)**
Cancer	36 (5.0)
Advanced adenoma	79 (10.9)
Non-advanced adenoma	66 (9.1)
Non-adenomatous polyp	52 (7.2)
Inflammatory disease	20 (2.8)
Vascular lesion	5 (0.7)
Others	3 (0.4)
**Total**	**261 (35.9)**

**Table 3:** Colonoscopy findings, FIT concentration and AUC according to study definitions.

	***n* (%)**	**FIT value μg/g (Q1-Q3)**	**AUC (95%CI)**
Any lesion^*^	261 (35.9)	18.9 (0.0–219.4)	0.703 (0.661–0.745)
Clinically significant pathology^+^	142 (19.5)	84.53 (3.8–1097.7)	0.813 (0.768–0.858)
Neoplasia	182 (25.0)	24.95(0.0–322.5)	0.724 (0.678–0.770)
Advanced neoplasia	115 (15.8)	75.55 (2.9–871.8)	0.791 (0.741–0.841)
Cancer	36 (5.0)	1079.05 (83.5–3178.4)	0.903 (0.848–0.957)

* *Any lesion is defined as presence of any finding in the colonoscopy [1 case defined as others (serrated polyp) is included]*.

+ *Clinically significant pathology is defined as presence of CRC, advanced adenoma, inflammatory bowel disease or vascular pathology [2 cases defined as others (serrated polyp and rectal ulcer) are included]*.

### Diagnostic Performance of FIT According to Colonoscopy Findings

FIT was positive in 25.9% of patients (188) using 20 μg/g as the cut-off value. Reducing the cut-off value to 10 μg/g, as suggested in some guidelines, increased the rate to 28.3% (206), which would have required a 9.6% increase in the number of colonoscopies (18). Ten of these 18 colonoscopies (55.6%) would have had a normal result. The other eight procedures (44.4%) would have had pathological results: five cases of advanced neoplasia (1 cancer, 4 advanced adenoma), one of microscopic colitis, one of vascular lesion and one case of non-advanced adenoma.

Three complications were recorded during the study: two perforations that required surgery and one case of post-polypectomy bleeding. FIT was negative (0 μg/g) in one of these cases and positive (≥20 μg/g of feces) in the other two.

Fecal hemoglobin concentration value increased as the lesions detected in the colonoscopies were more severe. The area under the curve (AUC) of FIT increased parallel to the severity of lesions (Table 3).

Sensitivity, specificity, PPV, and NPV were calculated for the different lesions using the standard cut-off value (20 μg/g) used in our area, obtaining a high NPV for cancer as well as for clinically significant pathology and advanced neoplasia. The diagnostic performance of FIT was also calculated using the cut-off value suggested in some guidelines (10 μg/g), with similar results for NPV and higher sensitivity but lower specificity (Table 4).

**Table 4:** Diagnostic performance of FIT according to different cut-off values.

**FIT ≥20 μg/g**	**TP**	**FP**	**TN**	**FN**	**Se (95%CI)**	**Sp (95%CI)**	**PPV (95%CI)**	**NPV (95%CI)**	OR^*^ (95%CI)
Any Lesion	127	61	405	134	48.7% (42.7–54.7)	86.9% (83.5–89.7)	67.6% (60.6–73.8)	75.1% (71.3–78.6)	5.8 (4.0–8.3)
Clinically significant pathology	97	91	494	45	68.3% (60.3–75.4)	84.4% (81.3–87.2)	51.6% (44.5–58.6)	91.7% (89.0–93.7)	10.7 (7.0–16.4)
Neoplasia	98	90	455	84	53.8% (46.6–60.9)	83.5% (80.1–86.4)	52.1% (45.0–59.2)	84.4% (81.1–87.2)	5.2 (3.6–7.7)
Advanced neoplasia	77	111	501	38	67.0% (57.9–74.9)	81.9% (78.6–84.7)	41.0% (34.2–48.1)	92.9% (90.5–94.8)	8.2 (5.2–12.9)
Cancer	33	155	536	3	91.7% (78.2–97.1)	77.6% (74.3–80.5)	17.6% (12.8–23.6)	99.4% (98.4–99.8)	31.4 (9.5–104.5)
**FIT ≥10 μg/g**	**TP**	**FP**	**TN**	**FN**	**Se (95%CI)**	**Sp (95%CI)**	**PPV (95%CI)**	**NPV (95%CI)**	OR^*^ (95%CI)
Any Lesion	135	71	395	126	51.7% (45.7–57.7)	84.8% (81.2–87.7)	65.5% (58.8–71.7)	75.8% (72.0–79.3)	5.4 (3.8–7.7)
Clinically significant pathology	104	102	483	38	73.2% (65.4–79.8)	82.6% (79.3–85.4)	50.5% (43.7–57.2)	92.7% (90.1–94.6)	11.8 (7.6–18.2)
Neoplasia	104	102	443	78	57.1% (49.9–64.1)	81.3% (77.8–84.3)	50.5% (43.7–57.2)	85.0% (81.7–87.8)	5.0 (3.5–7.4)
Advanced neoplasia	82	124	488	33	71.3% (62.5–78.8)	79.7% (76.4–82.7)	39.8% (33.4–46.6)	93.7% (91.2–95.5)	8.5 (5.4–13.6)
Cancer	34	172	519	2	94.4% (81.9–98.5)	75.1% (71.8–78.2)	16.5% (12.1–22.2)	99.6% (98.6–99.9)	41.7 (9.9–176.5)
***p*****-value**^**+**^									
Any lesion					**0.005**	**0.002**	0.081	0.112	
Clinically significant pathology					**0.008**	**0.001**	0.305	**0.021**	
Neoplasia					**0.014**	**0.001**	0.131	0.126	
Advanced neoplasia					**0.025**	**<0.001**	0.253	0.067	
Cancer					0.317	**<0.001**	0.071	0.350	

* *OR: risk of presenting the outcome with a positive FIT compared to a negative FIT. Adjusted by sex and age*.

+ *p value refers to the comparison of Se, Sp, PPV and NPV with the two cut-off values for the different outcomes*.

*Bold values highlight the statistically significant data*.

### Diagnostic Performance of FIT According to the Location of Colonoscopy Findings

Sensitivity, specificity, PPV, and NPV of FIT were calculated for advanced neoplasia and cancer according to their location in the colon using the two different cut-off values. No differences were observed according to the locations of these lesions and FIT performed very well, maintaining a high NPV using both cut-off values, with a slight decrease in specificity using the lowest value for all locations of lesions (Table 5). After reducing the cut-off value to ≥10 μg/g, the sensitivity of the test for detecting advanced neoplasia or cancer was similar in the rectum and right-sided colon, but clearly superior in the left-sided colon as compared to ≥20 μg/g.

**Table 5:** Diagnostic performance of FIT according to location and cut-off value.

**FIT ≥20 μg/g**	**TP**	**FP**	**TN**	**FN**	**Se (95%CI)**	**Sp (95%CI)**	**PPV (95%CI)**	**NPV (95%CI)**	OR^*^ (95%CI)
**Cancer**	33	155	536	3	91.7% (78.2–97.1)	77.6% (74.3–80.5)	17.6% (12.8–23.6)	99.4% (98.4–99.8)	31.4 (9.5–104.5)
Right-sided colon	10	178	538	1	90.9% (62.3–98.4)	75.1% (71.8–78.2)	5.3% (2.9–9.5)	99.8% (99.0–100.0)	22.5 (2.8–179.1)
Left-sided colon	10	178	537	2	83.3% (55.2–95.3)	75.1% (71.8–78.1)	5.3% (2.9–9.5)	99.6% (98.7–99.9)	11.4 (2.5–53.2)
Rectum	13	175	539	0	100.0% (77.2–100.0)	75.5% (72.2–78.5)	6.9% (4.1–11.5)	100.0% (99.3–100.0)	–
Left-sided colon + rectum	23	165	537	2	92.0% (75.0–97.8)	76.5% (73.2–79.5)	12.2% (8.3–17.7)	99.6% (98.7–99.9)	31.4 (7.3–135.3)
**Advanced neoplasia**	77	111	501	38	67.0% (57.9–74.9)	81.9% (78.6–84.7)	41.0% (34.2–48.1)	92.9% (90.5–94.8)	8.2 (5.2–12.9)
Right-sided colon	20	168	527	12	62.5% (45.3–77.1)	75.8% (72.5–78.9)	10.6% (7.0–15.9)	97.8% (96.1–98.7)	4.3 (2.0–9.1)
Left-sided colon	36	152	520	19	65.5% (52.3–76.6)	77.4% (74.1–80.4)	19.1% (14.2–25.4)	96.5% (94.6–97.7)	5.4 (3.0–9.8)
Rectum	21	167	532	7	75.0% (56.6–87.3)	76.1% (72.8–79.1)	11.2% (7.4–16.5)	98.7% (97.3–99.4)	8.4 (3.5–20.3)
Left-sided colon + rectum	57	131	513	26	68.7% (58.1–77.6)	79.7% (76.4–82.6)	30.3% (24.2–37.2)	95.2% (93.0–96.7)	7.5 (4.5–12.6)
**FIT ≥10 μg/g**	**TP**	**FP**	**TN**	**FN**	**Se (95%CI)**	**Sp (95%CI)**	**PPV (95%CI)**	**NPV (95%CI)**	OR^*^ (95%CI)
**Cancer**	34	172	519	2	94.4% (81.9–98.5)	75.1% (71.8–78.2)	16.5% (12.1–22.2)	99.6% (98.6–99.9)	41.7 (9.9–176.5)
Right-sided colon	10	196	520	1	90.9% (62.3–98.4)	72.6% (69.2–75.8)	4.9% (2.7–8.7)	99.8% (98.9–100.0)	19.3 (2.4–154.1)
Left-sided colon	11	195	520	1	91.7% (64.6–98.5)	72.7% (69.3–75.9)	5.3% (3.0–9.3)	99.8% (98.9–100.0)	21.9 (2.8–172.6)
Rectum	13	193	521	0	100.0% (77.2–100.0)	73.0% (69.6–76.1)	6.3% (3.7–10.5)	100.0% (99.3–100.0)	–
Left-sided colon + rectum	24	182	520	1	96.0% (80.5–99.3)	74.1% (70.7–77.2)	11.7% (8.0–16.8)	99.8% (98.9–100.0)	57.0 (7.6–426.9)
**Advanced neoplasia**	82	124	488	33	71.3% (62.5–78.8)	79.7% (76.4–82.7)	39.8% (33.4–46.6)	93.7% (91.2–95.5)	8.5 (5.4–13.6)
Right-sided colon	20	186	509	12	62.5% (45.3–77.1)	73.2% (69.8–76.4)	9.7% (6.4–14.5)	97.7% (96.0–98.7)	3.7 (1.7–7.8)
Left-sided colon	40	166	506	15	72.7% (59.8–85.7)	75.3% (71.9–78.4)	19.4% (14.6–25.4)	97.1% (95.3–98.2)	6.7 (3.6–12.6)
Rectum	22	184	515	6	78.6% (60.5–89.8)	73.7% (70.3–76.8)	10.7% (7.2–15.6)	98.8% (97.5–99.5)	8.9 (3.5–22.6)
Left-sided colon + rectum	62	144	500	21	74.7% (64.4–82.8)	77.6% (74.3–80.7)	30.1% (24.2–36.7)	96.0% (93.9–97.3)	8.8 (5.1–15.2)

* *OR: risk of presenting the outcome with a positive FIT compared to a negative FIT. Adjusted by sex and age*.

When FIT performance was stratified by sex and age, FIT performed slightly better in patients under the age of 60, but with similar figures for both cut-off values (Supplementary Tables 1, 2).

### Diagnostic Performance of FIT in Combination With Symptoms

Sensitivity, specificity, PPV, and NPV were calculated for FIT in combination with the main symptom that prompted the demand for colonoscopy. No combination of symptom plus FIT yielded better results than FIT alone. An increase in specificity was obtained with the combination of symptoms and FIT, but with a profound reduction in sensitivity and negative predictive value, using the two different cut-off values evaluated in the study (Tables 6–8). The combination of different symptoms such as anemia + rectal bleeding + FIT or abdominal pain + diarrhea + change in bowel habits + FIT did not improve the performance of FIT alone (data not shown).

**Table 6:** Diagnostic performance of FIT + symptom for colorectal cancer.

**FIT ≥20 μg/g**	**TP**	**FP**	**TN**	**FN**	**Se (95%CI)**	**Sp (95%CI)**	**PPV (95%CI)**	**NPV (95%CI)**	OR^*^ (95%CI)
FIT	33	155	536	3	91.7% (78.2–97.1)	77.6% (74.3–80.5)	17.6 % (12.8–23.6)	99.4% (98.4–99.8)	31.4 (9.5–104.5)
FIT + Rectal bleeding	15	48	643	21	41.7% (27.1–57.8)	93.1% (90.9–94.7)	23.8% (15.0–35.6)	96.8% (95.2–97.9)	11.4 (5.2–24.9)
FIT + Change in bowel habits	1	14	677	35	2.8% (0.5–14.2)	98.0% (96.6–98.8)	6.7% (1.2–29.8)	95.1% (93.2–96.4)	0.9 (0.1–7.6)
FIT + Diarrhea	0	24	667	36	0.0% (0.0–9.6)	96.5% (94.9–97.7)	0.0% (0.0–13.8)	94.9% (93.0–96.3)	–
FIT + Abdominal pain	2	20	671	34	5.6% (1.5–18.1)	97.1% (95.6–98.1)	9.1% (2.5–27.8)	95.2% (93.3–96.5)	1.6 (0.3–7.2)
FIT + Anemia	14	31	660	22	38.9% (24.8–55.1)	95.5% (93.7–96.8)	31.1% (19.5–45.7)	96.8% (95.2–97.9)	9.0 (4.1–20.1)
**FIT ≥10 μg/g**	**TP**	**FP**	**TN**	**FN**	**Se (95%CI)**	**Sp (95%CI)**	**PPV (95%CI)**	**NPV (95%CI)**	OR^*^ (95%CI)
FIT	34	172	519	2	94.4% (81.9–98.5)	75.1% (71.8–78.2)	16.5% (12.1–22.2)	99.6% (98.6–99.9)	41.7 (9.9–176.5)
FIT + Rectal bleeding	15	55	636	21	41.7% (27.1–57.8)	92.0% (89.8–93.8)	21.4% (13.4–32.4)	96.8% (95.2–97.9)	9.2 (4.3–19.9)
FIT + Change in bowel habits	2	19	672	34	5.6% (1.5–18.1)	97.3% (95.7–98.2)	9.5% (2.7–28.9)	95.2% (93.3–96.5)	1.5 (0.3–6.7)
FIT + Diarrhea	0	25	666	36	0.0% (0.0–9.6)	96.4% (94.7–97.5)	0.0% (0.0–13.3)	94.9% (93.0–96.3)	–
FIT + Abdominal pain	2	21	670	34	5.6% (1.5–18.1)	97.0% (95.4–98.0)	8.7% (2.4–26.8)	95.2% (93.3–96.5)	1.5 (0.3–6.9)
FIT + Anemia	14	32	659	22	38.9% (24.8–55.1)	95.4% (93.5–96.7)	30.4% (19.1–44.8)	96.8% (95.2–97.9)	8.7 (3.9–19.3)

* *OR: risk of presenting CRC with a positive FIT compared to a negative FIT. Adjusted by sex and age*.

**Table 7:** Diagnostic performance of FIT + symptom for advanced neoplasia.

**FIT ≥20 μg/g**	**TP**	**FP**	**TN**	**FN**	**Se (95%CI)**	**Sp (95%CI)**	**PPV (95%CI)**	**NPV (95%CI)**	OR^*^ (95%CI)
FIT	77	111	501	38	67.0% (57.9–74.9)	81.9% (78.6–84.7)	41.0% (34.2–48.1)	92.9% (90.5–94.8)	8.2 (5.2–12.9)
FIT + Rectal bleeding	31	32	580	84	27.0% (19.7–35.7)	94.8% (92.7–96.3)	49.2% (37.3–61.2)	87.3% (84.6–89.7)	7.4 (4.1–13.4)
FIT + Change in bowel habits	5	10	602	110	4.3% (1.9–9.8)	98.4% (97.0–99.1)	33.3% (15.2–58.3)	84.6% (81.7–87.0)	2.0 (0.6–6.4)
FIT + Diarrhea	7	17	595	108	6.1% (3.0–12.0)	97.2% (95.6–98.3)	29.2% (14.9–49.2)	84.6% (81.8–87.1)	2.7 (1.0–7.3)
FIT + Abdominal pain	6	16	596	109	5.2% (2.4–10.9)	97.4% (95.8–98.4)	27.3% (13.2–48.2)	84.5% (81.7–87.0)	1.8 (0.7–5.0)
FIT + Anemia	21	24	588	94	18.3% (12.3–26.3)	96.1% (94.2–97.4)	46.7% (32.9–60.9)	86.2% (83.4–88.6)	3.6 (1.8–6.9)
**FIT ≥10 μg/g**	**TP**	**FP**	**TN**	**FN**	**Se (95%CI)**	**Sp (95%CI)**	**PPV (95%CI)**	**NPV (95%CI)**	OR^*^ (95%CI)
FIT	82	124	488	33	71.3% (62.5–78.8)	79.7% (76.4–82.7)	39.8% (33.4–46.6)	93.7% (91.2–95.5)	8.5 (5.4–13.6)
FIT + Rectal bleeding	33	37	575	82	28.7% (21.2–37.5)	94.0% (91.8–95.6)	47.1% (35.9–58.7)	87.5% (84.8–89.8)	6.7 (3.8–11.7)
FIT + Change in bowel habits	7	14	598	108	6.1% (3.0–12.0)	97.7% (96.2–98.6)	33.3% (17.2–54.6)	84.7% (81.9–87.2)	2.4 (0.9–6.4)
FIT + Diarrhea	7	18	594	108	6.1% (3.0–12.0)	97.1% (95.4–98.1)	28.0% (14.3–47.6)	84.6% (81.8–87.1)	2.1 (0.8–5.5)
FIT + Abdominal pain	6	17	595	109	5.2% (2.4–10.9)	97.2% (95.6–98.3)	26.1% (12.5–46.5)	84.5% (81.7–87.0)	1.8 (0.7–4.8)
FIT + Anemia	21	25	587	94	18.3% (12.3–26.3)	95.9% (94.0–97.2)	45.7% (32.2–59.8)	86.2% (83.4–88.6)	3.4 (1.8–6.6)

* *OR: risk of presenting advanced neoplasia with a positive FIT compared to a negative FIT. Adjusted by sex and age*.

**Table 8:** Diagnostic performance of FIT + symptom for clinically significant pathology.

**FIT ≥20 μg/g**	**TP**	**FP**	**TN**	**FN**	**Se (95%CI)**	**Sp (95%CI)**	**PPV (95%CI)**	**NPV (95%CI)**	OR^*^ (95%CI)
FIT	97	91	494	45	68.3% (60.3–75.4)	84.4% (81.3–87.2)	51.6% (44.5–58.6)	91.7% (89.0–93.7)	10.7 (7.0–16.4)
FIT + Rectal bleeding	40	23	562	102	28.2% (21.4–36.1)	96.1% (94.2–97.4)	63.5% (51.1–74.3)	84.6% (81.7–87.2)	9.9 (5.6–17.6)
FIT + Change in bowel habits	6	9	576	136	4.2% (2.0–8.9)	98.5% (97.1–99.2)	40.0% (19.8–64.3)	80.9% (77.8–83.6)	2.3 (0.8–6.8)
FIT + Diarrhea	11	13	572	131	7.7% (4.4–13.3)	97.8% (96.2–98.7)	45.8% (27.9–64.9)	81.4% (78.3–84.1)	4.3 (1.8–10.4)
FIT + Abdominal pain	7	15	570	135	4.9% (2.4–9.8)	97.4% (95.8–98.4)	31.8% (16.4–52.7)	80.9% (77.8–83.6)	1.8 (0.7–4.6)
FIT + Anemia	23	22	563	119	16.2% (11.0–23.1)	96.2% (94.4–97.5)	51.1% (37.0–65.0)	82.6% (79.5–85.2)	9.7 (2.0–7.0)
**FIT ≥10 μg/g**	**TP**	**FP**	**TN**	**FN**	**Se (95%CI)**	**Sp (95%CI)**	**PPV (95%CI)**	**NPV (95%CI)**	OR^*^ (95%CI)
FIT	104	102	483	38	73.2% (65.4–79.8)	82.6% (79.3–85.4)	50.5% (43.7–57.2)	92.7% (90.1–94.6)	11.8 (7.6–18.2)
FIT + Rectal bleeding	43	27	558	99	30.3% (23.3–38.3)	95.4% (93.4–96.8)	61.4% (49.7–72.0)	84.9% (82.0–87.5)	9.1 (5.3–15.6)
FIT + Change in bowel habits	8	13	572	134	5.6% (3.0–10.7)	97.8% (96.2–98.7)	38.1% (20.8–59.1)	81.0% (78.0–83.7)	2.2 (0.9–5.4)
FIT + Diarrhea	11	14	571	131	7.7% (4.4–13.3)	97.6% (96.0–98.6)	44.0% (26.7–62.9)	81.3% (78.3–84.0)	3.9 (1.6–9.1)
FIT + Abdominal pain	7	16	569	135	4.9% (2.4–9.8)	97.3% (95.6–98.3)	30.4% (15.6–50.9)	80.8% (77.8–83.6)	1.7 (0.7–4.3)
FIT + Anemia	23	23	562	119	16.2% (11.0–23.1)	96.1% (94.2–97.4)	50.0% (36.1–63.9)	82.5% (79.5–85.2)	3.6 (1.9–6.7)

* *OR: risk of presenting clinically significant pathology with a positive FIT compared to a negative FIT. Adjusted by sex and age*.

### Risk Stratitication According to Fecal Hemoglobin Concentration and NNT

Patients that obtained a positive FIT result were classified in four groups, according to their fecal hemoglobin concentration. The risk of presenting advanced neoplasia, clinically significant pathology, and cancer adjusted by sex and age was calculated considering Q1 as the reference group. The risk was higher as the fecal hemoglobin concentration increased (Table 9).

**Table 9:** Risk of presenting clinically significant pathology, advanced neoplasia and cancer according to fecal hemoglobin concentration adjusted by sex and age.

	***p*-value**	**OR (95%CI)**
**Clinically significant pathology**		
Q1 (<47)		Reference
Q2 (47–110.6)	0.144	1.9 (0.8–4.5)
Q3 (110.6–1069.4)	0.004	3.6 (1.5–8.6)
Q4 (>1069.4)	<0.001	9.6 (3.7–24.9)
**Advanced neoplasia**		
Q1 (<47)		Reference
Q2 (47–110.6)	0.087	2.3 (0.9–5.8)
Q3 (110.6–1069.4)	0.030	2.8 (1.1–7.2)
Q4 (>1069.4)	0.001	4.9 (1.9–12.7)
**Cancer**		
Q1 (<47)		Reference
Q2 (47–110.6)	0.124	3.8 (0.7–20.2)
Q3 (110.6–1069.4)	0.075	4.5 (0.9–23.7)
Q4 (>1069.4)	0.001	16.3 (3.4–78.5)

*Bold values highlight the statistically significant data*.

NNTs were also calculated according to the two different cut-off values. NNTs were similar (3) when considering advanced neoplasia and clinically significant pathology for both cut-off values. NNT slightly increased when using to lower cut-off value (7) for cancer compared to 20 μg/g (6) (data not shown).

## Discussion

This study has evaluated the performance of FIT in patients with “red flag” symptoms that could suggest the presence of CRC or clinically significant pathology. FIT has shown a high NPV, not only for cancer, where it reached 99.4% [data consistent with prior studies (7–10)], but also for advanced neoplasia and clinically significant pathology. These findings should encourage the use of FIT in symptomatic patients before deciding on the need to perform an invasive test such as colonoscopy, since symptoms alone have poor specificity (6). The NPV of FIT in symptomatic patients may be of particular value compared to screening in asymptomatic patients, since here we need to identify patients who have no colonic pathology despite having abdominal symptoms, which may be useful for prioritizing colonoscopies when resources are limited, as is the case in most national public health systems.

In our study, <20% of participants presented clinically significant pathology. Using FIT in this cohort would potentially have avoided referral and subsequent invasive investigations in a high percentage of patients with no or mild pathology. Moreover, one of the complications recorded in the study would have been avoidable, since that patient presented a negative FIT result (0 μg/g).

FIT has also proven useful for prioritizing colonoscopies for symptomatic patients with positive results, especially those with high fecal hemoglobin concentrations in the test, as the risk of presenting a significant lesion is higher when the FIT hemoglobin concentration increases, which is consistent with prior studies (14–17). According to our data, FIT could be useful in two different ways: on the one hand, for avoiding unnecessary colonoscopies and potential complications in case of a negative FIT, which would help to decrease waiting lists, and on the other, for prioritizing endoscopies for patients with symptoms and a positive FIT result, especially those with higher fecal hemoglobin concentrations detected in the test (17). This would be especially helpful in health systems with a limited capacity for colonoscopies that are already overloaded with long waiting lists caused by the growing workload due to the implementation of CRC screening programmes and open access to primary care.

As more women than men participated in the study, a stratified analysis by sex and age was performed; FIT performance was similar according to sex, and only FIT performed a bit better in patients under 60.

Furthermore, in our study, the performance of FIT was evaluated using two different cut-off values: the one suggested in recent guidelines for symptomatic patients (10 μg/g) and the one used in multiple screening programmes worldwide, including our region (20 μg/g). Using the two different cut-off values, a similar NPV was obtained, with a decrease in specificity and a small increase in sensitivity when using the lower cut-off value. FIT performance was also evaluated for both cut-off values according to the location of colonoscopy findings, with a similar outcome. No significant differences in NPV or specificity values for FIT were observed between left-sided and right-sided lesions using either cut-off point.

According to our results, it may not be necessary to reduce the cut-off value used for symptomatic patients, and the standard value used in the screening programme should also be sufficient for the evaluation of symptomatic patients. It seems that reducing the cut-off value would increase the number of false positive results and consequently the number of colonoscopies, increasing already long waiting lists without any significant benefit.

A recent meta-analysis of studies evaluating FIT performance in CRC screening concludes that the sensitivity and specificity of detection of neoplastic colonic lesions vary with positive cut-off values and suggests that the cut-off value could be reduced at centers with sufficient resources for colonoscopy follow-up. A 49% increase in positive tests and, therefore, follow-up colonoscopies would be achieved by using a cut-off value of <10 μg/g rather than <20 μg/g, compared to 146% using a cut-off value >10 and <20 μg/g rather than >20 μg/g, which are the references used in our study for symptomatic patients (25). We are aware that although the performance of FIT with a cut-off of 20 μg/g had optimal outcomes, 45 patients presented clinically significant pathology in the colonoscopy and were FIT negative (35 advanced adenoma, 3 cancer, 2 vascular lesion, and 5 inflammatory bowel disease cases). Using the 10 μg/g cut-off value, 38 cases would have been FIT negative and would have presented clinically significant pathology (31 advanced adenoma, 2 cancer, 1 vascular lesion, and 4 inflammatory bowel disease cases), 28 of them with a FIT value of 0. Therefore, the difference between the two cut-off values evaluated (20 and 10 μg/g) is just 7 cases (1 cancer, 4 advanced adenoma, 1 inflammatory bowel disease, and 1 vascular lesion). The question now may be how to avoid missing cases (especially cancer) without paying the price of a higher rate of false positives, longer waiting lists, and loss of opportunity due to the delayed diagnosis of cancer in overloaded services. As FIT has the strength of being a user-friendly test, for symptomatic patients with a FIT value between 10 and 20 μg/g, repeating the test before deciding on the need for an invasive procedure would be an option, though more studies are needed prior making a recommendation. Another option could be combining FIT with another non-invasive test, such as calprotectin. In this line a recent study (26) showed the combination of FIT and fecal calprotectin as a cost-effective strategy to avoid colonoscopies in symptomatic patients without relevant pathology, but further research is needed in this field. Meanwhile, when suspicion of clinically significant pathology is high, even with a negative FIT result, a colonoscopy or other studies should be considered.

Finally, our study has also evaluated the performance of FIT combined with different symptoms, using the two different cut-off values. When FIT was combined with symptoms, we obtained a slight increase in specificity but a decrease in sensitivity and NPV with both cut-off points. According to our results, FIT alone, without taking into account the symptom that motivated the performance of the test, would be enough to decide on the need to perform subsequent invasive tests such as colonoscopy.

We are aware our study has limitations like the sample size, specially when symptoms are taken into account, since the subgroup of patients who meet these conditions is small in some cases, new studies with a larger sample size would be helpful to confirm our results.

## Conclusions

The specificity and NPV of FIT for clinically significant pathology, advanced neoplasia, and cancer are high in symptomatic patients. FIT is a helpful test for determining the need to perform further studies without taking symptoms into account. Reducing the cut-off value below 20 μg of Hb/g of feces for symptomatic patients may not be necessary in health systems with a limited capacity for follow-up endoscopies, since the performance of FIT using the standard cut-off value offers almost identical NPV. FIT can be used to avoid or prioritize colonoscopy procedures.

## Data Availability Statement

The datasets generated for this study are available on request to the corresponding author.

## Ethics Statement

The studies involving human participants were reviewed and approved by Regional Ethical Committee of Aragón (CEICA). The patients/participants provided their written informed consent to participate in this study.

## Author Contributions

MN collected data, analyzed data, and drafted the manuscript. GH, CS, and ALu collected data. PC-L analyzed data and performed all statistical analysis. JP-L performed biochemical analysis. ALa designed the study, analyzed data, and drafted the manuscript. All authors revised the manuscript and contributed to its intellectual content.

## Conflict of Interest

AL is Advisor to Sysmex Iberia (Barcelona, Spain), manufacturer of SENTiFIT 270 FOB Gold^®^. The remaining authors declare that the research was conducted in the absence of any commercial or financial relationships that could be construed as a potential conflict of interest.
