# Supplementary material for: Reducing the Cut-Off Value of the Fecal Immunochemical Test for Symptomatic Patients Does Not Improve Diagnostic Performance
Source: Front Med (Lausanne). 2020 Sep 2;7:410. doi: 10.3389/fmed.2020.00410 (PMC7492376; doi:10.3389/fmed.2020.00410)
Supplement: Supplementary file 1 [file Data_Sheet_1.PDF]

Suppl Table 1. Diagnostic performance of FIT according to sex and cut-off value.

FIT ≥ 20 μg/g	TP	FP	TN	FN	Se	Sp	PPV	NVP	OR* (95%CI)
**Lesion**	127	61	405	134	48.7% (42.7-54.7)	86.9% (83.5-89.7)	67.6% (60.6-73.8)	75.1% (71.3-78.6)	6.3 (4.4-9.0)
Male	66	23	168	65	50.4% (41.9-58.8)	88.0% (82.6-91.8)	74.2% (64.2-82.1)	72.1% (66.0-77.5)	7.4 (4.3-12.9)
Female	61	38	237	69	46.9% (38.6-55.5)	86.2% (81.6-89.8)	61.6% (51.8-70.6)	77.5% (72.4-81.8)	5.5 (3.4-9.0)
p for interaction									0.430
**Cancer**	33	155	536	3	91.7% (78.2-97.1)	77.6% (74.3-80.5)	17.6% (12.8-23.6)	99.4% (98.4-99.8)	38.0 (11.5-125.7)
Male	17	72	233	0	100.0% (81.6-100.0)	76.4% (71.3-80.8)	19.1% (12.3-28.5)	100.0% (98.4-100.0)	-
Female	16	83	303	3	84.2% (62.4-94.5)	78.5% (74.1-82.3)	16.2% (10.2-24.7)	99.0% (97.2-99.7)	19.5 (5.5-68.4)
p for interaction									-
**Advanced neoplasia**	77	111	501	38	67.0% (57.9-74.9)	81.9% (78.6-84.7)	41.0% (34.2-48.1)	92.9% (90.5-94.8)	9.1 (5.9-14.2)
Male	43	46	211	22	66.2% (54.0-76.5)	82.1% (77.0-86.3)	48.3% (38.2-58.5)	90.6% (86.1-93.7)	9.0 (4.9-16.4)
Female	34	65	290	16	68.0% (54.2-79.2)	81.7% (77.3-85.4)	34.3% (25.7-44.1)	94.8% (91.7-96.8)	9.5 (4.9-18.2)
p for interaction									0.902
**Neoplasia**	98	90	455	84	53.8% (46.6-60.9)	83.5% (80.1-86.4)	52.1% (45.0-59.2)	84.4% (81.1-87.2)	5.9 (4.1-8.5)
Male	54	35	190	43	55.7% (45.8-65.2)	84.4% (79.1-88.6)	60.7% (50.3-70.2)	81.5% (76.1-86.0)	6.8 (4.0-11.7)
Female	44	55	265	41	51.8% (41.3-62.1)	82.8% (78.3-86.6)	44.4% (35.0-54.3)	86.6% (82.3-90.0)	5.2 (3.1-8.7)
p for interaction									0.467
**Clinically significant pathology**	97	91	494	45	68.3% (60.3-75.4)	84.4% (81.3-87.2)	51.6% (44.5-58.6)	91.7% (89.0-93.7)	11.7 (7.7-17.8)
Male	50	39	208	25	66.7% (55.4-76.3)	84.2% (79.1-88.2)	56.2% (45.8-66.0)	89.3% (84.6-92.6)	10.7 (5.9-19.2)

Female	47	52	286	20	70.1% (58.3-79.8)	84.6% (80.4-88.1)	47.5% (37.9-57.2)	93.5% (90.1-95.7)	12.9 (7.087-23.6)
p for interaction									0.655
**FIT ≥ 10 μg/g**	**TP**	**FP**	**TN**	**FN**	**Se**	**Sp**	**PPV**	**NVP**	**OR* (95%CI)**
**Lesion**	135	71	395	126	51.7% (45.7-57.7)	84.8% (81.2-87.7)	65.5% (58.8-71.7)	75.8% (72.0-79.3)	6.0 (4.2-8.5)
Male	71	28	163	60	54.2% (45.7-62.5)	85.3% (79.6-89.7)	71.7% (62.2-79.6)	73.1% (66.9-78.5)	6.9 (4.1-11.7)
Female	64	43	232	66	49.2% (40.8-57.7)	84.4% (79.6-88.2)	59.8% (50.3-68.6)	77.9% (72.8-82.2)	5.2 (3.3-8.4)
p for interaction									0.447
**Cancer**	34	172	519	2	94.4% (81.9-98.5)	75.1% (71.8-78.2)	16.5% (12.1-22.2)	99.6% (98.6-99.9)	51.3 (12.2-215.7)
Male	17	82	223	0	100.0% (81.3-100.0)	73.1% (67.9-77.8)	17.2% (11.0-25.8)	100.0% (98.3-100.0)	-
Female	17	90	296	2	89.5% (68.6-97.1)	76.7% (72.2-80.6)	15.9% (10.2-24.0)	99.3% (97.6-99.8)	28.0 (6.3-123.3)
p for interaction									-
**Advanced neoplasia**	82	124	488	33	71.3% (62.5-78.8)	79.7% (76.4-82.7)	39.8% (33.4-46.6)	93.7% (91.2-95.5)	9.8 (6.2-15.3)
Male	47	52	205	18	72.3% (60.4-81.7)	79.8% (74.4-84.2)	47.5% (37.9-57.2)	91.9% (87.6-94.8)	10.3 (5.5-19.2)
Female	35	72	283	15	70.0% (56.3-80.9)	79.7% (75.2-83.6)	32.7% (24.6-42.1)	95.0% (91.9-96.9)	9.2 (4.8-17.7)
p for interaction									0.803
**Neoplasia**	104	102	443	78	57.1% (49.9-64.1)	81.3% (77.8-84.3)	50.5% (43.7-57.2)	85.0% (81.7-87.8)	5.8 (4.0-8.3)
Male	58	41	184	39	59.8% (49.8-69.0)	81.8% (76.2-86.3)	58.6% (48.7-67.8)	82.5% (77.0-86.9)	6.7 (3.9-11.3)
Female	46	61	259	39	54.1% (43.6-64.3)	80.9% (76.3-84.9)	43.0% (34.0-52.5)	86.9% (82.6-90.3)	5.0 (3.0-8.3)
p for interaction									0.443
**Clinically significant pathology**	104	102	483	38	73.2% (65.4-79.8)	82.6% (79.3-85.4)	50.5% (43.7-57.2)	92.7% (90.1-94.6)	13.0 (8.4-19.9)

Male	55	44	203	20	73.3% (62.4-82.0)	82.2% (77.0-86.5)	55.6% (45.7-65.0)	91.0% (86.6-94.1)	12.7 (6.9-23.3)
Female	49	58	280	18	73.1% (61.5-82.3)	82.8% (78.5-86.5)	45.8% (36.7-55.2)	94.0% (96.7-96.1)	13.1 (7.1-24.2)
p for interaction									0.936

*OR: risk of presenting the outcome with a positive FIT compared to a negative FIT. Adjusted by sex and age.

Suppl Table 2. Diagnostic performance of FIT according to age and cut-off value.

FIT ≥ 20 µg/g	TP	FP	TN	FN	Se	Sp	PPV	NVP	OR* (95%CI)
**Lesion**	127	61	405	134	48.7% (42.7-54.7)	86.9% (83.5-89.7)	67.6% (60.6-73.8)	75.1% (71.3-78.6)	6.3 (4.4-9.0)
<60 years	37	29	231	57	39.4% (30.1-49.5)	88.8% (84.4-92.1)	56.1% (44.1-67.4)	80.2% (75.2-84.4)	5.2 (2.9-9.1)
≥60 years	90	32	174	77	53.9% (46.3-61.3)	84.5% (78.9-88.8)	73.8% (65.3-80.8)	69.3% (63.4-74.7)	6.4 (3.9-10.3)
p for interaction									*0.587*
**Cancer**	33	155	536	3	91.7% (78.2-97.1)	77.6% (74.3-80.5)	17.6% (12.8-23.6)	99.4% (98.4-99.8)	38.0 (11.5-125.7)
<60 years	8	58	287	1	88.9% (56.5-98.0)	83.2% (78.9-86.7)	12.1% (6.3-22.1)	99.7% (98.1-99.9)	39.6 (4.9-322.6)
≥60 years	25	97	249	2	92.6% (76.6-97.9)	72.0% (67.0-76.4)	20.5% (14.3-28.5)	99.2% (97.1-99.8)	32.1 (7.5-138.1)
p for interaction									*0.872*
**Advanced neoplasia**	77	111	501	38	67.0% (57.9-74.9)	81.9% (78.6-84.7)	41.0% (34.2-48.1)	92.9% (90.5-94.8)	9.1 (5.9-14.2)
<60 years	16	50	280	8	66.7% (46.7-82.0)	84.8% (80.6-88.3)	24.2% (15.5-35.8)	97.2% (94.6-98.6)	11.2 (4.6-27.6)
≥60 years	61	61	221	30	67.0% (56.9-75.8)	78.4% (73.2-82.8)	50.0% (41.3-58.7)	88.0% (83.5-91.5)	7.4 (4.4-12.4)
p for interaction									*0.430*
**Neoplasia**	98	90	455	84	53.8% (46.6-60.9)	83.5% (80.1-86.4)	52.1% (45.0-59.2)	84.4% (81.1-87.2)	5.9 (4.1-8.5)
<60 years	21	45	258	30	41.2% (28.8-54.8)	85.1% (80.7-88.7)	31.8% (21.8-43.8)	89.6% (85.5-92.6)	4.0 (2.1-7.6)
≥60 years	77	45	197	54	58.8% (50.2-66.8)	81.4% (76.0-85.8)	63.1% (54.3-71.2)	78.5% (73.0-83.1)	6.2 (3.9-10.0)
p for interaction									*0.278*
**Clinically significant pathology**	97	91	494	45	68.3% (60.3-75.4)	84.4% (81.3-87.2)	51.6% (44.5-58.6)	91.7% (89.0-93.7)	11.7 (7.7-17.8)
<60 years	30	36	276	12	71.4% (56.4-82.8)	88.5% (84.4-91.5)	45.5% (34.0-57.4)	95.8% (92.9-97.6)	19.2 (9.0-40.7)

≥60 years	67	55	218	33	67.0% (57.3-75.4)	79.9% (74.7-84.2)	54.9% (46.1-63.5)	86.9% (82.1-90.5)	8.0 (4.8-13.4)
p for interaction									*0.062*
**FIT ≥ 10 μg/g**	**TP**	**FP**	**TN**	**FN**	**Se**	**Sp**	**PPV**	**NVP**	**OR* (95%CI)**
**Lesion**	135	71	395	126	51.7% (45.7-57.7)	84.8% (81.2-87.7)	65.5% (58.8-71.7)	75.8% (72.0-79.3)	6.0 (4.2-8.5)
<60 years	38	34	226	56	40.4% (31.1-50.5)	86.9% (82.3-90.5)	52.8% (41.4-63.9)	80.1% (75.1-84.4)	4.5 (2.6-7.8)
≥60 years	97	37	169	70	58.1% (50.5-65.3)	82.0% (76.2-86.7)	72.4% (64.3-79.3)	70.7% (64.7-76.1)	6.3 (4.0-10.1)
p for interaction									*0.357*
**Cancer**	34	172	519	2	94.4% (81.9-98.5)	75.1% (71.8-78.2)	16.5% (12.1-22.2)	99.6% (98.6-99.9)	51.3 (12.2-215.7)
<60 years	8	64	281	1	88.9% (56.5-98.0)	81.4% (77.0-85.2)	11.1% (5.7-20.4)	99.6% (98.0-99.9)	35.1 (4.3-285.8)
≥60 years	26	108	238	1	96.3% (81.7-99.3)	68.8% (63.7-73.4)	19.4% (13.6-26.9)	99.6% (97.7-99.9)	57.3 (7.7-425.7)
p for interaction									*0.741*
**Advanced neoplasia**	82	124	488	33	71.3% (62.5-78.8)	79.7% (76.4-82.7)	39.8% (33.4-46.6)	93.7% (91.2-95.5)	9.8 (6.2-15.3)
<60 years	16	56	274	8	66.7% (46.7-82.0)	83.0% (78.6-86.7)	22.2% (14.2-33.1)	97.2% (94.5-98.6)	9.8 (4.0-24.0)
≥60 years	66	68	214	25	72.5% (62.6-80.6)	75.9% (70.6-80.5)	49.3% (40.9-57.6)	89.5% (85.0-92.8)	8.3 (4.9-14.2)
p for interaction									*0.759*
**Neoplasia**	104	102	443	78	57.1% (49.9-64.1)	81.3% (77.8-84.3)	50.5% (43.7-57.2)	85.0% (81.7-87.8)	5.8 (4.0-8.3)
<60 years	21	51	252	30	41.2% (28.8-54.8)	83.2% (78.5-87.0)	29.2% (19.9-40.5)	89.4% (85.2-92.4)	3.5 (1.8-6.5)
≥60 years	83	51	191	48	63.4% (54.8-71.1)	78.9% (73.4-83.6)	61.9% (56.5-69.7)	79.9% (74.4-84.5)	6.5 (4.0-10.4)
p for interaction									*0.119*
**Clinically significant pathology**	104	102	483	38	73.2% (65.4-79.8)	82.6% (79.3-85.4)	50.5% (43.7-57.2)	92.7% (90.1-94.6)	13.0 (8.4-19.9)

<60 years	31	41	271	11	73.8% (58.9-84.7)	86.9% (82.7-90.2)	43.1% (32.3-54.6)	96.1% (93.2-97.8)	18.6 (8.7-39.9)
≥60 years	73	61	212	27	73.0% (63.6-80.7)	77.7% (72.4-82.2)	54.5% (46.0-62.7)	88.7% (84.1-92.1)	9.4 (5.6-15.9)
p for interaction									*0.147*

*OR: risk of presenting the outcome with a positive FIT compared to a negative FIT. Adjusted by sex and age.
